# Microfluidics in Haemostasis: A Review

**DOI:** 10.3390/molecules25040833

**Published:** 2020-02-14

**Authors:** Heta Jigar Panchal, Nigel J Kent, Andrew J S Knox, Leanne F Harris

**Affiliations:** 1 School of Biological and Health Sciences, Technological University Dublin (TU Dublin) - City Campus, Kevin Street, Dublin D08 NF82, Ireland; d18126564@mydit.ie (H.J.P.); andrew.knox@TUDublin.ie (A.J.S.K.); 2 engCORE, Faculty of Engineering, Institute of Technology Carlow, Kilkenny Road, Carlow R93 V960, Ireland; nigel.kent@itcarlow.ie

**Keywords:** coagulation, haemostasis, LOC, MEMS, microfluidics, POC

## Abstract

Haemostatic disorders are both complex and costly in relation to both their treatment and subsequent management. As leading causes of mortality worldwide, there is an ever-increasing drive to improve the diagnosis and prevention of haemostatic disorders. The field of microfluidic and Lab on a Chip (LOC) technologies is rapidly advancing and the important role of miniaturised diagnostics is becoming more evident in the healthcare system, with particular importance in near patient testing (NPT) and point of care (POC) settings. Microfluidic technologies present innovative solutions to diagnostic and clinical challenges which have the knock-on effect of improving health care and quality of life. In this review, both advanced microfluidic devices (R&D) and commercially available devices for the diagnosis and monitoring of haemostasis-related disorders and antithrombotic therapies, respectively, are discussed. Innovative design specifications, fabrication techniques, and modes of detection in addition to the materials used in developing micro-channels are reviewed in the context of application to the field of haemostasis.

## 1. Introduction

Haemostasis is a complex wound healing process, activated by nearly eighty biochemical reactions that arrest blood at the site of injury while maintaining normal blood flow elsewhere in a vascular system [1]. A haemostatic imbalance can lead to either excessive bleeding or undesired clotting conditions. It is expected that, by 2030, the treatment cost for blood coagulation related disorders will rise to approximately USD 800 billion [2]. Complex vascular networks and blood flow parameters can be modelled using microfluidics to aid the understanding of the pathophysiology of blood disorders [3]. Broadly, microfluidics refers to the handling and manipulation of, typically sub millilitre, volumes of fluids. The particular physical laws governing fluids at this level have led to new areas of research with many diverse applications [4]. Lab on a chip (LOC) technology, largely enabled through microfluidics, is a multidisciplinary subject dedicated to the development of laboratory experiments carried out in a miniaturised format. The miniaturised nature of these LOC devices typically results in the design and manufacture of fluidic networks with sub millimetre characteristic dimensions [5]. Among the major advantages of microfluidic and LOC technology in the field of biology and biochemistry is its application to high-throughput screening (HTS). HTS is now a well-established technology used primarily in drug discovery. With an increase in the number of compounds and molecular targets available, major pharmaceutical and biotechnology companies are using HTS technologies such as robotics, lab automation, optimised detectors, etc. to screen 10,000–100,000 compounds daily [6]. The screening of compounds can be achieved with cost-effective miniaturisation technology, which not only reduces the consumption of reagents but also efficiently manages parallel sample-processing in a shorter period compared to alternative conventional methods [7].

Thrombosis is the leading cause of morbidity and mortality, responsible for approximately one in four deaths worldwide, based on the statistics derived from the Global Burden of Diseases study from 1990–2010 [8,9]. Thrombus (clot) formation is the result of an interaction of platelets at the site of injury and the meshwork around the platelets formed by fibrin (fibre-like protein) deposition. Arterial thrombosis results in the formation of platelet-rich clots in the presence of a high shear rate, whereas venous thrombosis is the result of fibrin-rich clots occurring at low shear rates. The dynamic shear rate driving the blood flow in a complex vascular network of varied dimensions is a crucial factor responsible for both venous and arterial thrombosis [10]. Micro-fabrication technology has created new horizons for novel designs in microfluidic devices that can imitate diverse physiologically relevant structures required for the investigation of multi-factorial causes of thrombosis [11]. With the progressions in protein adhesion techniques in microfluidic devices, the biomimetic micro-channels can be internally coated with appropriate biological substrates, such as von Willebrand factor (VWF), collagen, Tissue Factor (TF) or fibrinogen. Furthermore, these micro-channels can be designed to attain shear stresses ranging from low venous to high arterial, resembling in vivo conditions within the same device for examining the role of platelet adhesion during thrombosis [12].

The global market share for antithrombotic drugs is 53.1% of all the drugs for cardiovascular disease (CVD) [13]. Typically, antithrombotic drugs, which are broadly categorized as anticoagulants and antiplatelets drugs, require continuous monitoring to reduce the risk of excessive bleeding or clotting [14]. Aspirin is the first line of treatment as an antiplatelet drug while warfarin and heparin are the commonly prescribed anticoagulants in cardiovascular therapy [15]. Conventional clot-based assays include prothrombin time (PT), activated partial thromboplastin time (aPTT), thrombin clotting time (TCT), and activated clotting time (ACT). These assays are used for evaluating associated coagulopathic conditions and monitoring antithrombotic therapy. In laboratory settings, a PT test is performed by measuring the clotting time after adding a TF reagent and calcium to the plasma. The prolongation in PT is converted to the international normalised ratio (INR) and facilitates monitoring of PT/INR [16]. Miniaturised point of care (POC) devices for home monitoring and self-testing are hugely popular due to their rapid turnaround time and low sample volume requirement [17,18].

Microfluidic devices developed specifically for whole blood detection assays can reduce the sample preparation time and provide rapid results. This reduction in time from sample to result is highly desirable in patients with traumatic conditions, including critical illness, perioperative haemorrhage and severe blood loss due to coagulation abnormalities [19]. The need for rapid results with reduced turnaround time and microlitre volumes of samples have promoted the use of POC testing devices in the past few decades [20]. Micro-coagulation systems such as the ACT are employed in near-patient settings to regulate the heparin dosage during cardiopulmonary bypass surgery. The ACT test is carried out by introducing a small amount of whole blood into a cartridge preloaded with a coagulation activator such as celite, kaolin, glass beads, or phospholipids mixture and the clotting time is derived either mechanically, optically or electrochemically [21].

Most anticoagulants, including heparin and its derivative, low molecular weight heparin (LMWH), direct factor Xa (FXa), and thrombin inhibitors, are monitored using aPTT or specific chromogenic/fluorogenic coagulation assays. The aPTT assay was developed to monitor the intrinsic and common pathways of coagulation and is derived by adding a surface activator with phospholipid to the plasma sample. The average clotting time obtained from an aPTT assay is in the range of 22 to 40s [14,16]. Innovative approaches in centrifugal microchips and electro-mechanical detection systems have been developed as high-throughput microfluidic devices that consume microlitre volumes of sample whole blood and reagents to carry out aPTT tests in near-patient settings [22,23]. Chromogenic anti FXa assays are also carried out as an alternative to aPTT to measure the concentration of LMWH and FXa inhibitors based on the enzymatic cleavage of FXa specific substrates in laboratory settings. Harris et al. developed a microfluidic device incorporated with a miniaturised fluorogenic anti-FXa assay specifically designed to conduct the quantitative assessment of heparin using a fluorescence detection system [24]. Such advances in microfluidic technologies are providing a platform to develop portable, less labour-intensive and cost-effective devices that can be employed in near-patient settings as well as for the remote monitoring of CVD [25].

In this review, advanced microfluidic devices are discussed based on their innovative design specifications, fabrication techniques, and different modes of detection developed for the diagnosis of haemostasis related disorders and monitoring of antithrombotic therapies. The preferred range of materials that are used in developing micro-channels are presented and their significance in assay development is highlighted.

## 2. Microfluidic Chip Design

Microfluidics offers technology to enable the miniaturisation of biological assays, significantly impacting high-throughput drug screening applications. The micro-channels are generally small in cross sectional area, with governing dimensions ranging from micrometres to nanometres. This small feature size enables the ability to run many samples in parallel. This parallelisation, in conjunction with the small volumes required for a given test, facilitates high-throughput screening [26]. However, given the relatively small volumes of fluids required per test, the viscous forces present in the fluid typically dominate over the inertial forces. This results in a relatively unique set of design considerations. The efficient design of a microfluidic device is dependent upon the interaction of a number of factors relating to fluid dynamics at a micro scale. The performance of micro and nano-fluidic devices relies on an understanding of parameters including fluid velocity, flow rate, pressure gradient, diffusion coefficients and chemical interactions within the often-complex network of micro-channels [27]. In this section, the significant design features in accordance with mechanical parameters that are responsible for appropriate transport of the fluid and accurate mixing of reagents are reviewed.

### 2.1. Fluid Specific Considerations

#### 2.1.1. Miscible and Immiscible Fluids

In biological applications, an important consideration while designing a microfluidic device is creating an environment for appropriate mixing of reagents with samples. The fluid flow in the small channels of a microfluidic device is typically in the laminar regime. A characteristic of laminar flow is that the mixing of reagents and samples is governed by diffusion rates of the media. This process is slow relative to the turbulent flow regimes typical in macro-fluidic systems [28]. Different approaches for achieving the optimized mixing of fluids include various techniques to increase fluid–fluid interface area, modification of surface chemistries, magnetic stirring, differential pressure, and generating heat or electric field [29].

**Figure 1:**
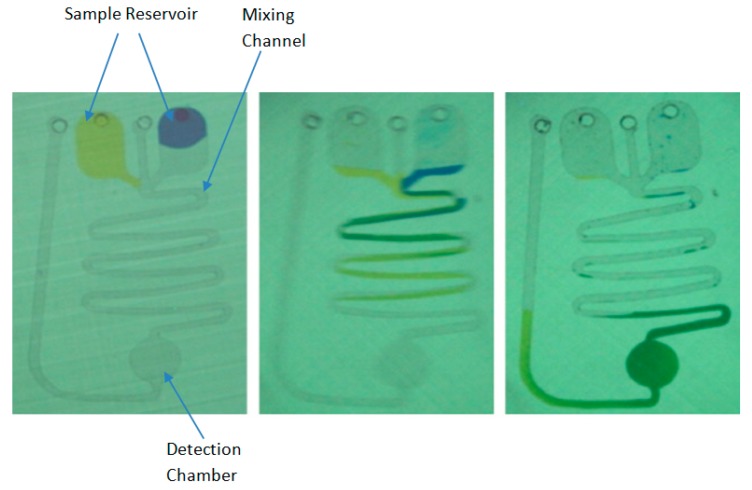
Microfluidic disc with two sample reservoirs and one mixing channel. After injecting samples into the reservoir in an uneven manner and applying centrifugal force, the sample with more volume starts flowing faster, and the uniform mixing is achieved in the long mixing channel. Taken with permission from [30].

Centrifugal-type microfluidic platforms have become popular in recent years. The characteristic nature of a centrifugal platform is that fluid flow is generated through a pressure differential caused by rotating the platform at defined speeds. In one such example, a microfluidic disk analyser (MDA) device was developed to execute the PT test. The device included two sample reservoirs, one for the application of human plasma and another for the PT reagent. On rotating the platform at a speed of 5000 rpm for two seconds, efficient mixing was achieved in a 200 mm long serpentine channel. Figure 1 shows the design of the reservoirs and the micro-channels in which the degree of mixing can be characterised by the change in colour, as both yellow and blue solutions progress along the serpentine ‘mixing’ channel towards the detection chamber [30]. With the capability of executing 15 parallel tests and 10 µl of sample with reagent, it proved advantageous compared to the gold standard benchtop analyser. The gold standard Sysmex CA-1500 cannot perform parallel testing and requires an order of magnitude more sample and reagent for a single test.

**Figure 2:**
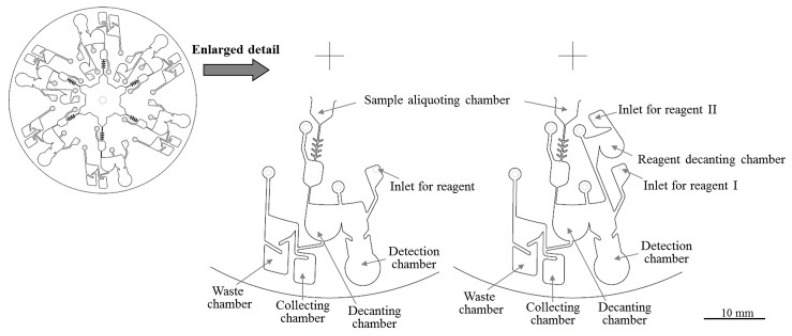
Microfluidic device design with six chambers to carry out three prothrombin time (PT) requiring one reagent inlet and three activated partial thromboplastin time (aPTT) assays that require inlets for reagents I and II. Taken with permission from [23].

Another application of centrifugal microfluidics for mixing and decanting includes assays such as the aPTT, where the requirement for two reagents complicates the structure of any device. This particular microfluidic centrifugal disk, shown in Figure 2, was designed to carry out three PT and three aPTT tests in parallel using whole blood. On application of whole blood to the aliquoting chamber located at the centre of the device (Figure 2), this multifaceted design separates plasma from whole blood using centrifugation. The plasma is then collected in a separate decanting chamber where it is mixed with the assay reagents to carry out further analysis [23].

**Figure 3:**
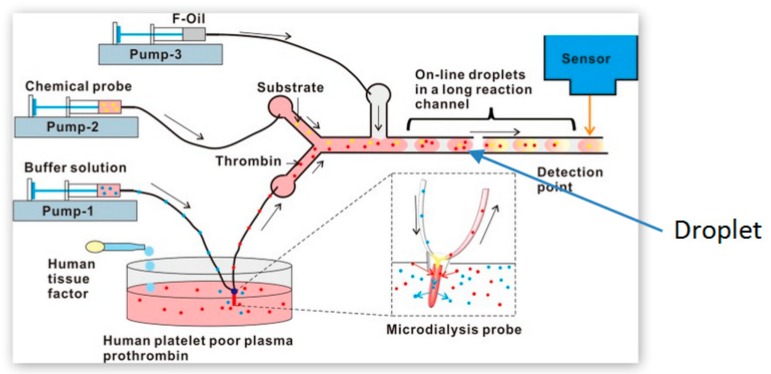
Schematic representation of droplet-based microfluidic device with a long channel for continuous analysis of thrombin generation in real-time. Taken with permission from [33].

An approach used to enable increased throughput in a microfluidic device involves the generation of droplets or plugs within a microchannel. These droplets are generated through the introduction of a fluid, immiscible with the assay sample and reagents, into the microchannel. Given the immiscible nature of the additional fluid, the sample and reagent are confined within a droplet, in turn preventing the dispersion of the sample and reagent throughout the microchannel [31]. This containment of the assay within a droplet, coupled with the ability to generate significant numbers of droplets for a given experiment, results in the capacity to generate a significant number of assays on a single microfluidic device. A microfluidic system to carry out aPTT measurement was developed using this ‘droplet microfluidic’ method. The developed device was comprised of different regions, including the droplet plug forming region, the mixing and merging zone and the detection region. The design included a winding channel with bends to induce chaotic mixing of the sample with the aPTT reagent within the plug and maintain the flow rate throughout the channel [32]. The same principle, but with a different channel design, was used in a diagnostic application for measuring real-time thrombin generation. As shown in Figure 3, a straight channel was made long enough to provide sufficient time for the proper mixing of an enzymatic reaction in each of the droplets before detection [33]. The invention of droplet-based technology in microfluidics has largely eliminated problems related to dispersion, contamination, and unnecessary adsorption to the walls of the channel.

#### 2.1.2. Shear Stress and Shear Rate

In an attempt to replicate physiological environments for blood flow in small blood vessels, microvascular structures can be mimicked using microfluidic devices. These microdevices consist of small arteriole-/venule-sized channels with vascular cells adhered to the walls of the device. The vasculature on a chip provides an ideal physiological environment to study different cell interactions, blood rheology in physiologically relevant channel sizes and the biochemical and biophysical processes responsible for multiple haemostasis-related disorders [34]. Two important parameters that are particularly taken into consideration while designing micro vasculatures for blood flow are shear rate and shear stress [35]. Shear stress is exerted on the walls of blood vessels by blood flow depending on flow rate and viscosity. Shear rate is a function of the velocity, defined as the rate of change in velocity of each layer in a laminar flow with respect to an adjacent layer [36,37]. Shear stress plays a vital role in thrombosis as demonstrated using various computational and mechanical models [38].

**Figure 4:**
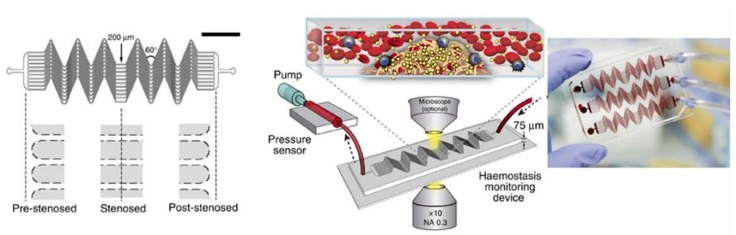
A biomimetic microfluidic device with angular bends designed to represent different shear rates at the diseased regions of the vascular system including a diagram of the device showing the stenosed, pre-stenosed and post-stenosed regions of the microchannel, a schematic of the haemostasis monitor and device and a photograph of three devices in a PDMS mold on a glass substrate. Taken with permission from [39].

A 12-channel arteriole-modelled microfluidic device was developed to monitor real-time clotting as well as platelet function for global assessment of haemostasis. The width of each of the 12 parallel channels was 200 micrometers, precisely designed to mimic a series of small blood vessels (arterioles) as shown in Figure 4. The microchannels were designed with alternating 60° bends and straight sections to enhance clot formation by providing maximum surface area to the sample blood. In addition to this, a part of the microchannels also represented the stenosed regions of blood vessels imitating the physiological narrowing in some vascular sections. The varying input blood flow rate resulted in high shear gradient and the formation of different sized clots within the microchannel. The shear-dependent clot formation occurring at angular micro-channels facilitated an understanding of the relationships between clotting time and the shear rate of the vessel wall at a controlled blood flow rate [39].

**Figure 5:**
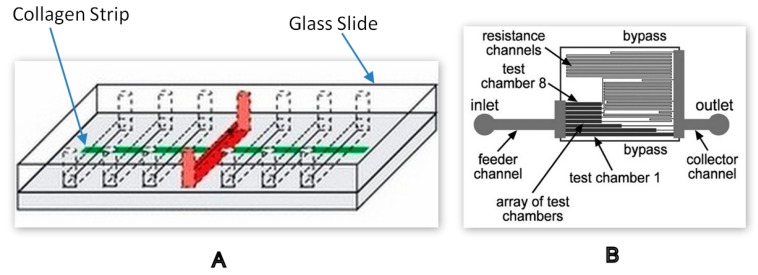
(**A**) Microfluidic device with 13 channels mounted on a glass slide patterned with a collagen strip [40]. (**B**) Schematic representation of a microfluidic device comprised of 8 parallel test chambers. Taken with permission from [12].

A shear-specific microfluidic device was developed to study the role of platelets during coagulation. The experimental device in this study for measuring murine platelet deposition used nearly 100 µL of blood volume from genetically modified mice. The device design comprised of 13 individual microchannels, each with a cross-section of 80 µm height and 100 µm width, as shown in Figure 5A, was fabricated using polydimethylsiloxane (PDMS). A thin film of collagen of 100 µm width was patterned on a glass slide and the PDMS device was mounted perpendicular to the collagen strip on the slide. The syringe pump was used to inject blood at different flow rates into the device and the non-constant shear rate was generated throughout the channel due to its cross-sectional geometry [40]. During the same year, another microfluidic chip was also developed to study shear-dependent platelet adhesion. The novel architecture in these devices included a resistance channel connected to each of the eight test chambers, as shown in Figure 5B. These resistance channels enabled a tunable pressure drop across each test chamber for a fixed inlet flow rate. The net effect of this tunable pressure drop was that the shear rate in each test chamber could be tuned to physiologically relevant shear rates in response to proteins such as collagen, fibrinogen and VWF factor, which are responsible for platelet activation and coagulation [12]. The fabrication of microvasculature replicas on a chip to study shear-dependent haemostasis-related disorders has made it possible to run multiple tests with minimal blood volume. The decreased turnover time for the tests compared to standard laboratory methods further demonstrates the high-throughput applications of microfluidics.

#### 2.1.3. Viscosity

**Figure 6:**
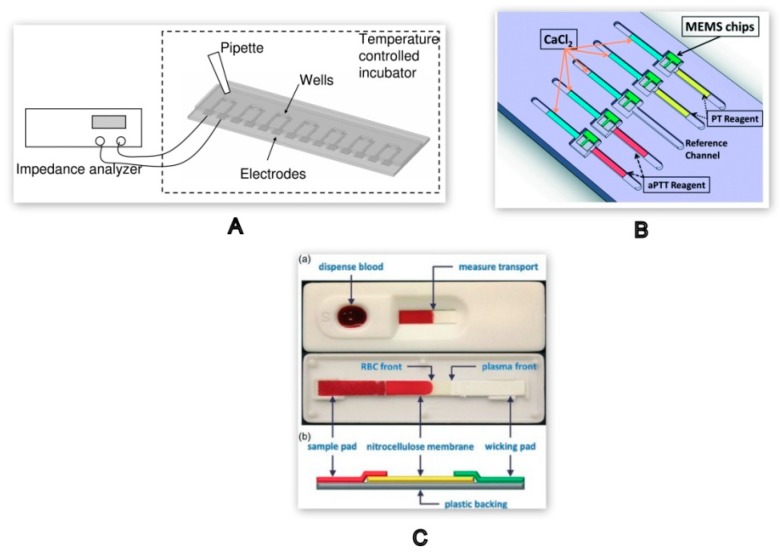
(**A**) High-throughput microfluidic chip with seven parallel wells for measuring blood coagulation time [42]. (**B**) MEMS-based microfluidic device [43]. (**C**) Schematic representation of a paper-based microfluidic chip. Taken with permission from [44].

Rate of blood flow in the vascular system is a function of viscosity. As the blood coagulates, there is a major change in its viscoelastic properties [41]. Blood clotting time can be measured by monitoring the change in viscosity due to the formation of a fibre-like protein, fibrin, during coagulation. A high-throughput microfluidic device comprised of seven sample wells has been designed to determine blood coagulation time using blood plasma resistance as a key parameter. The aggregation of red blood cells and fibrin formation in the blood clot will result in an increase in blood viscosity and solidification of blood plasma. Each well in the microchip was incorporated with an electrode that can run seven parallel tests, as shown in Figure 6A. The blood sample of only 10 µL was required to obtain the blood clotting time [42].

A combination of Micro-ElectroMechanical Systems (MEMS) and microfluidics was used to develop a chip based on micro-cantilevers to carry out both PT and aPTT in the same cartridge. The device was comprised of an array of 2 µm thick nickel micro-cantilevers bonded to the microfluidic channels, as shown in Figure 6B. The blood viscosity was monitored based on the changes in phase and amplitude of the resonant frequency. By monitoring the blood viscosity of 10 µL of the sample, the coagulation time for both PT and aPTT was derived. The result thus obtained correlated with the data obtained from CoaguChek XS, a commercially available POC device [43].

A paper-based microfluidic lateral flow test strip, shown in Figure 6C, was designed to derive the blood clotting time from the viscosity of whole blood in the presence of CaCl_2_ (calcium chloride). A blood sample of 10–20 µL, when added to the inlet reservoir, flows through the microchannel by means of capillary action. The relative surface energies of the blood constituents result in the plasma being separated from the red blood cells (RBCs). The travel time of the RBCs on the strip decreases upon increasing the concentration of CaCl_2_ resulting in rapid blood coagulation. The clotting time obtained was verified using the commercially available coagulation analyser CoaData 2000 Fibrintimer^®^ [44].

#### 2.1.4. Additional Design Specifications

In one distinctive application, a microfluidic device was coupled with nano-liquid chromatography (nano-LC) and mass spectrometry to detect inhibitors of two significant enzymes in coagulation, FXa and thrombin. This high-resolution screening technique was aimed at identifying enzyme inhibitors in a precise and rapid manner. The chip of approximately 45mm in length was divided into two microreactors. The microchannels of the first micro-reactor provided an environment to carry out the reaction of the enzyme-inhibitor reaction, which was quantified in the presence of a fluorogenic substrate in the second microreactor. This system provided better screening of the compounds when compared to the traditional multi-well plate method [45].

Microfluidic device designs can be utilised to create a biological model that can imitate physiological functions. One such biomimetic microfluidic model comprising all three blood vessels, an artery, a vein, and few capillaries was designed to represent the human circulatory system. The model was used to demonstrate the self-repair process in haemostasis with the help of non-biochemical reactions [46]. A novel design concept of introducing a venous valve in a micro-tubular structure allowing blood flow and clot propagation has also been implemented into a microfluidic device that was developed to study the intricate haemostasis mechanism [1]. Such creative designs are providing a platform on which to develop innovative multi-assay near-patient devices for application in the field of haemostasis.

### 2.2. Transduction Methods

LOCs are gaining popularity with their ability to perform multiple reactions in a single microfluidic device. The advancements in microfabrication technologies allow for the integration of innovative LOC devices coupled with efficient detection systems. The nature of the approach used for microfluidic detection plays a crucial role in improvising the system in terms of its responsiveness, accuracy, sensitivity, cost-effectiveness, and portability [47,48,49].

Optical detection techniques using spectroscopic methods such as absorbance, fluorescence and chemiluminescence are widely used in biological microfluidic applications. Laser-induced fluorescence (LIF) is a highly sensitive and well-established detection technique for the analysis of various fluorescence-emitting biomolecules in high throughput microfluidic devices. With advancement in fabrication techniques for integrating microelectrodes within devices, several methods are used in biomedical applications to detect different electrical parameters based on applied current/voltage. The efficient and low-cost production of micro-electrodes has resulted in significant use of electrochemical techniques [50,51,52]. Another common detection technique used in microchips designed for application to proteomics is the integration of the outlet with mass spectrometry (MS) [53]. Apart from the methods above, other transduction approaches include Nuclear Magnetic Resonance (NMR), Surface Plasmon Resonance (SPR), Raman spectroscopy, and acoustic wave-dependent detection, incorporated in micro- and nano-fluidic devices [48,53].

Blood clot formation is a result of a series of biochemical reactions occurring simultaneously that involves many particles, cells, proteins and other blood components in the process. There are a range of assays developed for monitoring antithrombotic therapies and diagnosing underlying haemostatic disorders. These assays are designed in accordance with particular blood components specific to their application. Micro-analytical devices based on these assays either have integrated sensors [54] or separate detection systems based on the electrical [55], acoustic [56], mechanical [57], rheological [58] or optical [59] properties of coagulated blood.

#### 2.2.1. Electrical Impedance Dependent Detection Techniques

Since 1948, it has been demonstrated by a series of experiments that blood clotting causes continuous changes in blood impedance. The change in impedance is related to the fibrinogen concentration in plasma. The formation of fibrin and platelet accumulation is directly proportional to an increase in impedance during the initial stage of coagulation, whereas, in the later phase of clot retraction, there is a decrease in the impedance value [60,61,62]. Electrochemical impedance can be used to accurately determine the clotting time of blood under controlled voltage and temperature conditions [63,64].

**Figure 7:**
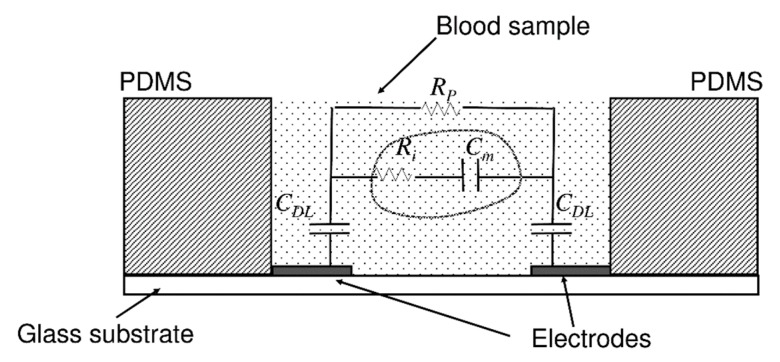
Electrical model representing three major blood components responsible for blood impedance, Rp – plasma resistance, Ri – Red blood cell interior resistance and Cm – Cell membrane capacitance. Taken with permission from [42].

A high-throughput microfluidic device developed using the principle of electrical impedance to analyse the blood coagulation time at varied temperature and haematocrit conditions was based on the electrical model as shown in Figure 7. The equivalent circuit for measuring the blood impedance consists of four major components: the plasma resistance (R_P_), red blood cell interior resistance (R_i_), cell membrane capacitance (C_m_), and double-layer capacitance of the electrode (C_DL_). On application of 0.1V across the electrodes, the changes in blood impedance in terms of phase angle and amplitude are continuously monitored. R_P_ and R_i_ play a vital role in intensifying the impedance over the clotting duration whereas the Cm dominates the impedance at the initial stages of coagulation [42]. During the same year, another POC device for monitoring aPTT in the presence of an anticoagulant was fabricated with interdigitated electrodes (IDE). During clot formation the comb like structure of IDE is useful in minimizing the leakage current. The signal from the electrodes was used to measure coagulation time in whole blood with varying heparin concentrations [22]. Commercially available devices such as anti-platelet and INR monitors, including INRatio 2 PT^®^, PlateletWorks^®,^ and Multiplate^®^, all use electrical impedance-based detection. These devices work on whole blood assays. Using whole blood assays is advantageous, as it reduces the sample preparation thus increasing the throughput. Typical test times for these commercial devices is approximately 2–10 min [65,66].

#### 2.2.2. Optical Detection Method

##### Microscopic Methods

**Figure 8:**
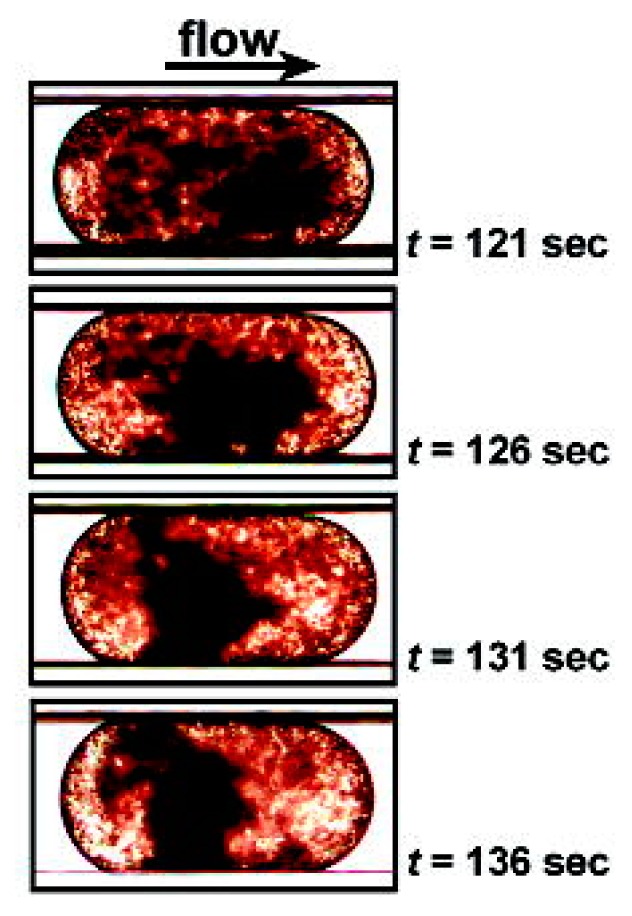
Bright-field microscopic image analysis of a single plug representing the formation of the clot through a dark spot with respect to time. Taken with permission from [32].

Fluorescence microscopy is the most preferable detection technique used in crucial studies of biomolecules and cellular components, as well as living cells [67]. Recently, Paiè et al. [68] published a detailed review article describing advancements in the fluorescence microscopic techniques used in next generation microfluidic devices newly termed as Microscopes-on-Chip (MOC). Bright-field microscopy enabled with high-speed cameras is one of the most common detection techniques used to study the physical properties and dynamic behaviour of droplets formed in the micro-channels [49]. An application of bright-field microscopy in combination with fluorescence microscopy was employed to study the effect of argatroban, a direct thrombin inhibitor, on aPTT in a plug based microfluidic device. A series of images of a single plug travelling throughout the micro-channel were captured using bright-field microscopy and the quantitative measurement of thrombin generation was carried out using fluorescence microscopy. The analysis of the collective images displayed a clot in the form of a dark spot in the middle of the plug resulting from red blood cells (RBCs) suspended within the fibrin trap, as shown in Figure 8 at the 136 s time interval. aPTT was derived from the time taken to obtain the dark spot as well as the increase in fluorescence intensity with high thrombin production during coagulation [32].

In one study, time-lapse fluorescence microscopy was used to demonstrate the dynamic behaviour of blood clot formation throughout an entire length of a micro-channel mimicking diseased vascular regions. The results obtained after the continuous monitoring of thrombus formation using fluorescently labelled fibrinogen and platelet imaging could be correlated to in vivo thrombus formation in stenosed (narrowed) arterioles. These results were further processed to obtain the clotting time [39]. The microfluidic channel network in the biomimetic microfluidic device was fabricated using PDMS. This channel network was then sealed to a standard microscope cover slide. The dynamics of platelet aggregation under flow through this channel network could then be interrogated using epi-fluorescence microscopy. The image processing of fluorescently labelled platelets demonstrated the progression of platelet aggregation similar to in vivo micro-vascular networks and also concluded that the device could be used for analysis of platelets in varying shear rate environments [69]. The fluorescence microscopic detection method has been used by several groups to study shear rate-dependent analysis of blood components including platelets, tissue factor, and complex vascular deformation during thrombosis, using novel designs in microfluidic devices [34,40,70,71,72].

##### Non-Microscopic Optical Methods

The cost-effective detection of bio-analytes is becoming increasingly popular, with leading edge techniques enabling the integration of optical detectors in microfluidic devices. Novel developments in fabricating the nano- and microdevices with lens-less microscope detection systems was discussed in depth by Pires et al. This is achieved by employing optical sensors, including complementary metal-oxide-semiconductors (CMOSs), charged-coupled devices (CCDs), lasers and filters [47]. Smartphone cameras and desktop scanners have made it possible to capture and transmit almost real-time images from an off-site centre to the experts for analysis. This ability to capture and store images for analysis is becoming increasingly popular. One area where this is particularly true is where colorimetric assays are employed. CMOSs, typically used in smart phone type devices, have significantly improved their signal-to-noise ratio in recent years, so much so that both quantitative and qualitative assays can be carried out [73].

**Figure 9:**
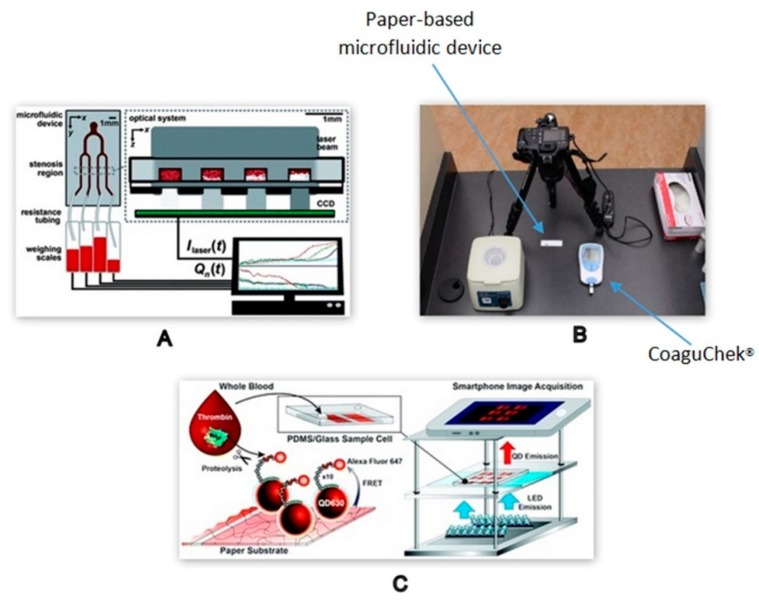
(**A**) Charged-coupled device (CCD) optical detection of the laser beam transmitted to the microfluidic device developed for a platelet aggregation study [74]. (**B**) Experimental set-up showing a digital camera mounted over the paper-based microfluidic device to capture images every 15s during the test [75]. (**C**) Smartphone based detection system of a paper-on-polydimethylsiloxane (PDMS) device using LED emission light. Taken with permission from [76].

Li et al. demonstrated a microscope-free detection technique to study platelet aggregation at multiple shear rates by integrating a laser diode, filters, and CCD sensor in a micro-device fabricated with PDMS, as depicted in Figure 9A. The results, in terms of increase in light intensity during platelet aggregation using this novel optical detection system, were highly correlated with the outcomes of conventional microscopic measurements [74]. A simple, cost-effective paper-based microfluidic device was also developed to monitor international normalised ratio (INR) in patients on warfarin using the high-resolution images captured by a digital camera set up, as shown in Figure 9B. The digital camera captured images every 15s for the entire test run time of 4 min. The images were digitally processed using ImageJ to obtain the distance travelled by RBCs. The correlation between the distance travelled by RBCs and INR was established and further validated with the results obtained from the commercially available POC CoaguChek^®^ device. The novel test strip presented a qualitative interpretation of INR results in terms of ‘Low,’ ‘OK,’ and ‘High’ [75].

A different application of a smartphone detection system is schematically represented in Figure 9C. In this system, a paper-on-PDMS device was fabricated to immobilise quantum dots (QDs) conjugated to a fluorophore (Alexa Fluor 647-maleimide) labelled peptide substrate specific for thrombin. Upon excitation with LEDs, the fluorescence emitted following the cleavage of fluorophore in the presence of thrombin was captured by the smartphone camera. The variations in fluorescence intensity were processed in the form of a series of images to evaluate the amount of thrombin in whole blood [76]. A high-throughput microfluidic paper analytical device (µPAD) comprised of eight channels was developed to determine the concentration of direct thrombin inhibitors in the presence of the ecarin clotting chromogenic assay (ECA) using smartphone camera images as a mode of detection. The observations from the processed images demonstrated an elevated absorbance value when correlated with the standard spectrometer measurements, with ambient lighting conditions a possible causative factor [77]. It is envisaged that with more sophisticated image processing techniques, any discrepancy due to ambient lighting conditions would be eradicated.

#### 2.2.3. Mechanical Detection Methods

**Figure 10:**
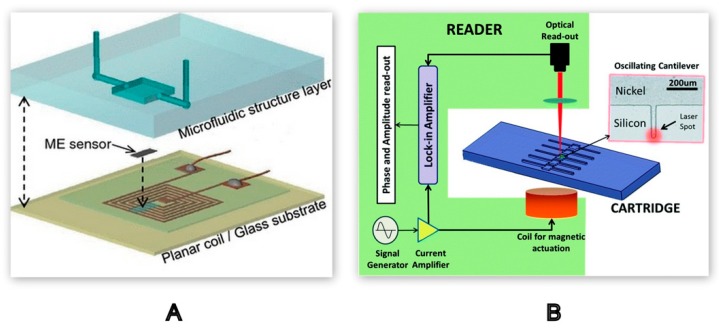
(**A**) Placement of components in a microfluidic device working on the principle of resonant frequency [80]. (**B**) Working set-up of the microfluidic cartridge. Taken with permission from [43].

Blood coagulation leads to an increase in blood viscosity. This mechanical parameter can be analysed using MEMS-based mechanical sensors, often using resonance frequencies to infer the blood clotting time [78,79]. This principle was demonstrated in a microfluidic device integrated with a magnetoelastic (ME) sensor and a planar coil to measure blood plasma viscosity in real-time. Magnetoelastic sensors made of magnetoelastic materials provide non-contact actuation and monitoring of the resonance frequency. Upon the activation of the sensor in the presence of a magnetic field, it will produce vibrations resulting in magnetic flux which will be sensed by the pick-up coil surrounding the sensor. As shown in Figure 10A, the detection unit, consisting of a planar coil (7 mm × 7 mm), collected the resonant frequency of the ME sensor in the form of a spectrum that was further analysed to derive blood plasma viscosity, and the results were in the range of normal blood plasma viscosity [80].

A LOC sensor with a separate detection system is schematically represented in Figure 10B. The design and development of this micro device was specific to avoid any direct electrical contact, as the cantilevers were actuated using a magnetic coil. Upon actuation, the resonant peak of vibration of the cantilever will vary as coagulation progresses. The study was conducted with multiple blood coagulation tests to obtain blood clotting time using the resonant frequency generated by the dynamic micro-cantilever system with a laser doppler vibrometer as a detection unit [43].

## 3. Microfluidic Device Fabrication

### 3.1. Materials for Device Fabrication

Microfabrication technologies began as a result of the significant drive towards the miniaturisation of integrated circuits in the microelectronics industry. As such, these technologies were typically focused on silicon materials. As these technologies transitioned to LOC-type devices, they were applied to glass due to the similarities in properties with silicon and the fact that existing laboratory techniques were well established using glassware. Both silicon and glass have some desirable mechanical properties such as high thermostability, solvent compatibility and stable surface charge. Further technologies are well established to enable construction of nano-channels less than 100 nm [81,82]. A major limitation of silicon and glass devices is the typical cost of manufacture at mass production volumes. Other challenges, such as issues around the disposal of glass-based devices, have resulted in alternative materials such as polymers, elastomers, plastics, and paper being used to fabricate LOC devices [83]. With the introduction of these new materials came additional fabrication techniques.

Among the more common materials used in the development of microfluidic and high throughput coagulation related devices is polydimethylsiloxane (PDMS). This material has become favourable due to its optical transparency, low-temperature curing, non-toxicity, reversible deformity, and smoothness [84,85]. The high porosity of PDMS also facilitates air permeability, a potentially desirable factor for biological applications including cell culture-mediated devices [86]. One such example of a microfluidic device using eight parallel test chambers to study the importance of platelet adhesion in haemostasis was developed using PDMS. The selection of PDMS as a material for this study was based on its property of non-specific adsorption and ease in fabrication, where it was observed that blood platelets attach to only specific factors in the blood but do not adhere to the walls of PDMS device [12]. Zhang et al. developed a microfluidic device to demonstrate the reduction in thrombus formation during blood flow experiments. This was achieved by modifying the surface properties of PDMS through polymerisation with sulfobetaine monomer. The modified PDMS showed minimal attachment of plasma proteins, hence reducing clot formation within the device. This technique could be implemented in scenarios where unobstructed whole blood movement is desired, such as dialysis [87].

PDMS is widely used in prototype microfluidic device fabrication. Fabrication is typically carried out using a soft lithography technique where the high compliance of the elastomer has made it possible to develop complex microstructures. Though PDMS has been widely used, there are some limitations. PDMS is limited in terms of its use with all organic solvents, restricting its use in some applications [82]. The low stiffness of PDMS can lead to micro-channel deformation, making it unsuitable for high-pressure fluidic applications. An undesirable absorption of molecules in PDMS further adds to its limitations in various cell culture-based micro-devices [88,89]. Although the process of modifying PDMS resulting in compatibility with a desired application may overcome these limitations, researchers also utilise other materials to enable LOC devices. Among the more common of these materials are thermoplastics.

Thermoplastics are widely used in industrial applications because of their easy availability and cost-effective mass production. Polymethylmethacrylate (PMMA), polyvinyl chloride (PVC), cyclic olefin polymer (COC/COP) and polystyrene (PS) are some of the thermoplastics that exhibit good mechanical strength, rigidity, thermal stability, and relative chemical/biological inertness, and are commonly used in microfluidic devices [90]. The large-volume manufacturing of micro-devices using plastic can be achieved by well-established fabrication techniques such as injection moulding and casting, which further adds to its marketability [91]. Significant work has been carried out on a range of thermoplastics in terms of surface modifications to promote specific binding of a given target molecule. Given the mechanical robustness and relative ease of fabrication, additional functionality such as pumps, valves and sensors can, in some cases, be incorporated as part of the LOC device itself [82]. A PMMA-based microfluidic dielectric sensor device was designed and fabricated to monitor whole blood coagulation time using the principle of dielectric spectroscopy (DS). DS was used to monitor the temporal variation in the permittivity of blood as a function of frequency. Sensing electrodes deposited on the biomedical grade PMMA measured the permittivity of blood during the clotting phase using only 9µl of the sample blood [92].

Over the past decade, there has been a surge in paper-based microfluidics, which have significant potential for application to biomedical diagnostics [93]. Paper contains cellulose as a key component and possesses advantages in terms of its distinct properties, such as being lightweight, inexpensive, readily accessible and can be chemically modified to enhance its biological compatibility for integrating different functional groups to it [94,95]. Recently, a paper-based diagnostic micro-device was developed to study blood viscosity and coagulation time that costs less than 50 cents per device [75]. A unique combination of paper on PDMS, as discussed earlier, was used to develop a microfluidic device for a POC application. The nitrocellulose paper was modified using a series of reactions for the immobilisation of quantum dots with peptide conjugates and fluorophore [76].

Material selection plays an enormous role in driving the fluid mechanisms of a microfluidic device. An appropriate selection of a material for the desired application is conditional to several factors, including flexibility, biological and chemical compatibility and surface properties [81,96]. While inherent mechanical properties of the material aid in the selection of the microchip fabrication technique, the optical/electrical properties of the material determine the type of detection method to be used.

### 3.2. Fabrication Techniques

Fabrication approaches are highly influenced by the type of material used to develop a device and the inherent properties of that material. Semiconductor materials like silicon-based devices can be fabricated using photolithography [97], which can further be used to fabricate polymeric devices using casting, injection molding [98], embossing, and imprinting techniques [99], while glass-based devices are produced by deep UV photolithography [100] and wet and dry etching methods [101]. With an increase in demand for disposable microfluidic devices in near-patient settings, cost-effective technologies, such as soft lithography, for developing polymeric micro and nano-chips have increased in the past two decades [102,103]. Laser-based micromachining techniques are also used for fabricating low-cost devices from thermoplastics [104]. In the past few years, µPADs, also termed as microfluidic paper-based analytical devices, have been gaining popularity because of their convenient accessibility and affordability. Solid wax and inkjet printing are high-throughput fabrication techniques that can significantly scale up the micro-patterning of µPADs [105,106].

#### 3.2.1. Direct Machining

##### Photolithography

**Figure 11:**
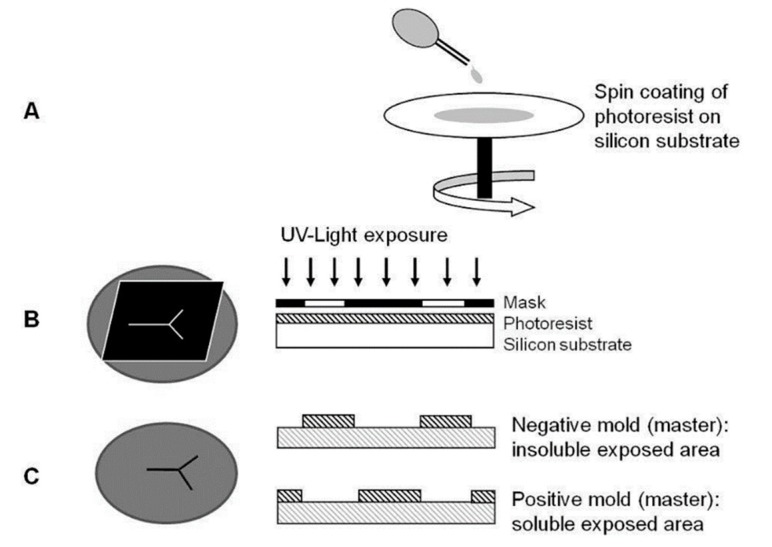
Schematic representation of the photolithographic fabrication technique. (**A**) A photoresist is spin-coated onto a silicon substrate. (**B**) The substrate with the spin-coated layer of photoresist is exposed to UV light through a high-resolution mask. (**C**) After baking and chemical development, the non-cross-linked material is removed, resulting in either a positive or a negative mold. Taken with permission from [111].

Photolithography is a common technique used in the development of microfluidic devices. It is a type of lithography that uses light to project a pre-designed pattern on to the desired material [107]. The first step in microfabrication with photolithography is to construct a photomask from the pattern of desired microchannels and typically created using design software [100]. Photomasks, generally made of quartz, are developed using techniques such as electron beam lithography and laser beam ablation [108,109]. The solid substrate, such as silicon or glass, is coated with a layer of photoresist. A photoresist is a photosensitive polymer responsible for the formation of shape and depth of microchannels [110]. In the presence of an ultraviolet (UV) light source, the pattern on a photomask is then transferred to the photoresist coated over the substrate. The final step involves the baking and chemical treatment of the substrate to develop the master template. The positive or negative moulds of the substrate can be obtained depending on the type of photoresist used in the fabrication process, as depicted in Figure 11 [111].

SU-8 is an epoxy-based photoresist material widely used in microfluidic chip development. It can be spin-coated to form a layer over the substrate that will outline the microchannel of desired depth and pattern upon application of UV energy through the photomask [110,112,113]. SU-8 UV photolithography was used to create a microvascular structure on silicon wafers to study the mechanical properties and geometry of blood vessels during thrombosis and other vascular diseases [34].

##### Chemical Etching

Silicon and glass patterning for the development of microfluidic devices can also be carried out through chemical etching. Chemical etching performed in the presence of a strong acid is referred as wet etching. Dry etching is carried out by reactive-ion etching (RIE) in the presence of plasma generated through an electromagnetic field. The reactive ions formed by plasma will carry physical etching to the exposed surface and form micro-patterns on glass as well as silicon. The process of dry etching is comparatively more time consuming as it is dependent on multiple factors, including plasma density, selected gas composition, applied voltage, temperature gradients generated on the substrates, and pressure in the source chamber [113,114,115].

**Figure 12:**
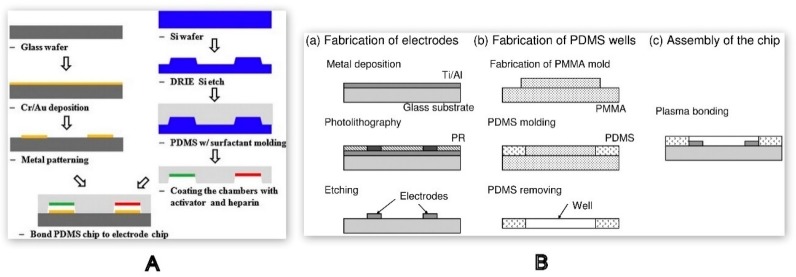
(**A**) Illustration of the fabrication process of a microfluidic chip using a glass wafer for electrode deposition and a silicon wafer for PDMS microchannel formation [22]. (**B**) Fabrication process for an electrochemical impedance-based blood coagulation device using photolithography. Taken with permission from [42].

Microchannel formation in conjunction with electrode integration within the microfluidic devices can be conducted with high precision through chemical etching combined with photolithography. This method involves standard photolithography followed by wet metal etching in the presence of an acid, such as hydrofluoric acid (HF) or hydrochloric acid (HCL). Cr/Au is one of the most effective etching layers for the micro-patterning of glass that can withstand high acidic conditions without forming any defects on the substrate [113,116,117]. A POC microfluidic device for measuring aPTT of whole blood using the principle of change in electrical impedance was integrated with 100 µm-wide Cr/Au electrodes using a wet etching technique. The fabrication of the device was carried out using two different molds, one based on glass wafer for electrode deposition and other based on a silicon wafer for microchannel formation using PDMS, and they were bonded together using thermal treatment, as shown in Figure 12A. This microfluidic device with a channel height of 100 µm was developed using a deep reactive ion etching (DRIE) technique. DRIE was used to create deep penetration by selective etching of the sacrificial layer of the silicon to develop the master template [22].

Figure 12B illustrates the fabrication of the electrodes for an electrical impedance-based blood coagulation monitoring device. Initially a Ti/Al layer is deposited onto the glass substrate. The layer is then coated with a photoresist which is selectively cured via a mask, indicated by the dark regions in Figure 12B (a) Photolithography. The uncured photoresist is then washed away, and the exposed Ti/Al substrate is etched, leaving only Ti/Al patterns under the cured photoresist. Finally, the cured resist is removed, and the final electrode pattern will stay on the glass substrate [42]. In addition to the aforementioned devices, a microfluidic dielectric sensor for obtaining blood clotting time using whole blood permittivity was fabricated with Ti/Au electrodes. An indirect etching technique defined as a lift-off process was employed to integrate the electrodes on selected regions of PMMA in which the Ti/Au was sputtered after the etching process [92].

##### Injection Moulding and Laser Processing

Thermoplastics are relatively easy to fabricate compared to the conventional silicon or glass-based devices and are more robust than elastomers such as PDMS. Injection moulding is a straightforward technique now being applied to fabricate micro- and nanofluidic devices [99]. In the injection moulding process, granules of a given thermoplastic are fed into the injection barrel and heated to beyond the melting point of the given thermoplastic. The melted material is injected into the pre-designed mould at relatively high pressure and allowed to cool down to take the shape of the desired micro-device [98,118]. Rapid prototyping of thermoplastics can be also be carried out by laser micromachining. This technology can produce a number of microfluidic devices of different designs without the requirement for master moulds [104]. A laser beam projected towards a given substrate such as thermoplastic, metal, polymer, ceramic or glass creates a cavity either photo-chemically or photo-thermally or by the combination of both. The precisely controlled fabrication of microchannels can be achieved by different fine-tuning parameters like laser power, moving speed of the laser beam, and distance between the substrate and the laser system [119].

**Figure 13:**
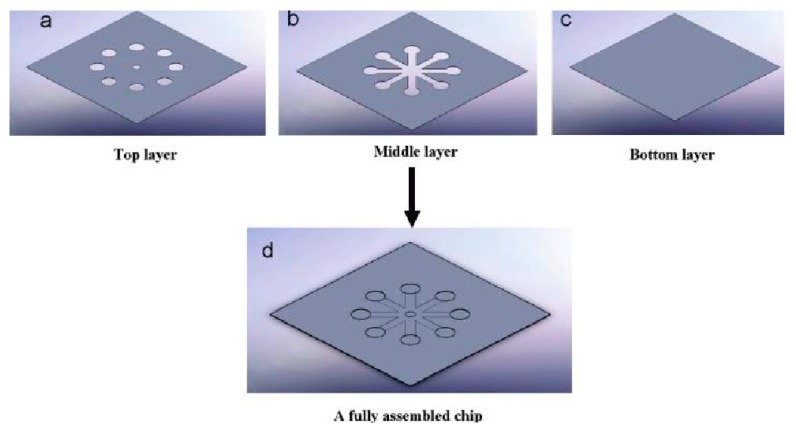
A high throughput eight chamber microfluidic chip composed of polymethylmethacrylate (PMMA), polycarbonate PC and pressure sensitive adhesive (PSA) and manufactured using laser micromachining The top layer (**a**), middle layer (**b**), and bottom layer (**c**) are assembled into a composite chip in (**d**). Taken with permission from [120].

An example of the fabrication and assembly of a high throughput microfluidic device is shown in Figure 13. This device was made of PMMA, PC and a double adhesive tape. The microchannels on the double adhesive tape were patterned using laser micromachining technique and it was adhered to the PMMA and PC layers. The device was fixed with permanent magnets in the bottom to capture the magnetic beads in the thrombin detection assay. Two thrombin binding aptamers were coated with magnetic beads and QDs, respectively. The detection of thrombin in the blood from the mixture of the other coagulation proteins was carried out by detecting the fluorescence generated by QDs. The permanent magnet was used to retain the thrombin attached to the magnetic beads during several washing steps throughout the entire assay [120]. Another microfluidic disk was fabricated using PMMA and polyethylene terephthalate (PET) to analyse PT using 14 µL of whole blood. Injection moulding was used to develop a 600 µm thick PMMA disk which was bonded with the PET lid using pressure sensitive adhesive (PSA). The centrifugal microfluidic disk could separate platelet poor plasma (PPP) from whole blood in the first stage of the test assay. The PPP thus obtained reacted with the PT reagent in the second stage to carry out further INR measurements [121].

##### Laminate-based and Printing Techniques

Among the simplest methods for fabricating microfluidic devices is to bind independent layers together by means of lamination. The overall steps for manufacturing laminated devices include the selection of the material, formation of desired features on individual layers through conventional methods such as cutting, machining or laser processing and subsequent bonding of appropriately aligned layers in a cleanroom type facility [122]. Based on the properties of the selected material, the binding can be performed using either an adhesive or a thermal mode of bonding. Thermal bonding is conducted by applying pressure to the layers that are pre-heated just beyond their glass transition temperature (Tg). Paper-based microfluidic analytical devices are developed using thermal bonding lamination, where the paper-microchip is sealed between the polyester lamination films when processed through the heated laminator [123]. Low cost and higher scalability are the primary advantages of the laminated-based production of micro and nanodevices.

The fabrication of biological analytical devices based on paper-based microfluidics technology started more than a decade ago, using approaches like photolithography, laser treatment, plasma treatment, etching techniques, and several printing methods including wax printing and inkjet printing. Li et al. (2012) discussed these techniques in detail in a paper-based microfluidic review article [105,124]. Cellulose fibres in paper are hydrophilic, on which selective hydrophobic reagents can be added to create a pattern of hydrophilic-hydrophobic micro-channels throughout the entire chip length. The hydrophobic boundary of the microchannel confines the sample and reagents and leads to the capillary movement within the channel [125]. The printing methods for fabrication of µPADs include heated hydrophobic solid wax deposition in wax printing [126], polystyrene impregnation on paper in flexographic printing, deposition of electrodes, and the alteration of the chemical composition of paper using alkyl ketene dimer (AKD) in inkjet printing [127].

**Figure 14:**
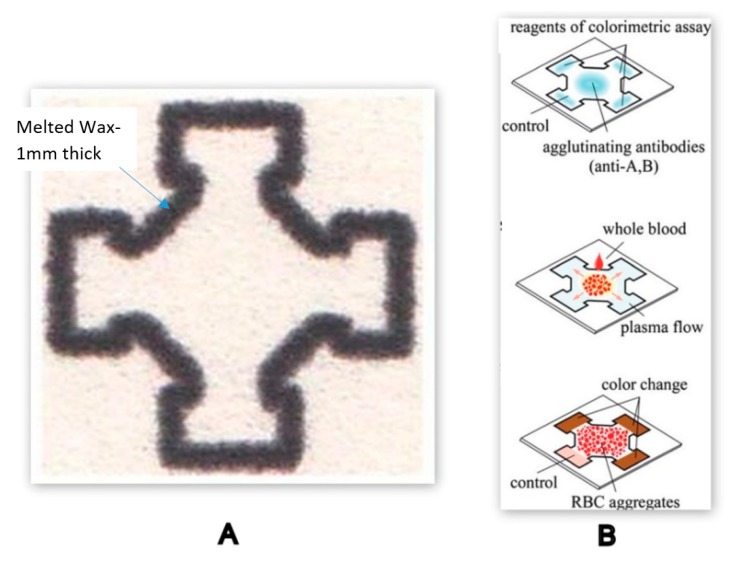
(**A**) Solid wax-printed microfluidic chip for separation of plasma from whole blood. (**B**) Schematic representation of a working µPAD. Taken with permission from [128].

Wax printing is an inexpensive and quick method that can produce approximately 100-200 µPADs in a single batch, making it suitable for various analytical and diagnostic biomedical applications [126]. A solid wax printer was used to fabricate a µPAD designed for separating plasma from whole blood. The solid wax was melted on the chromatography paper, creating a 1mm thick hydrophobic wall throughout the test region, as shown in Figure 14. The separation procedure involved the agglutination of RBCs in the centre spotted with antibodies (anti-A,B) and the transportation of plasma using capillary action in the lateral zones of the paper strip [128]. This simple microfluidic device could be used to develop plasma-based blood coagulation monitoring assays which will further reduce the laboratory sample preparation stages.

#### 3.2.2. Indirect Machining

Direct machining techniques can be used to fabricate the entire device or generate master templates (mould) that can be further processed through indirect methods such as casting, moulding, and embossing to produce efficient high throughput LOC devices. Hot embossing is a common method used for developing microfluidic channel networks for LOC devices. The basic steps in hot embossing involve heating the substrate polymer and master template at an elevated temperature, typically between Tg and the melting point of the material. A force is then applied between the master template and the polymer to emboss the structures existing on the master into the polymer. On the cooling of the polymer, these structures remain in the material. Thermoplastics are hot-embossed using metal or silicon masters, as they can withstand high temperatures and have significant longevity to replicate significant numbers of parts [99].

**Figure 15:**
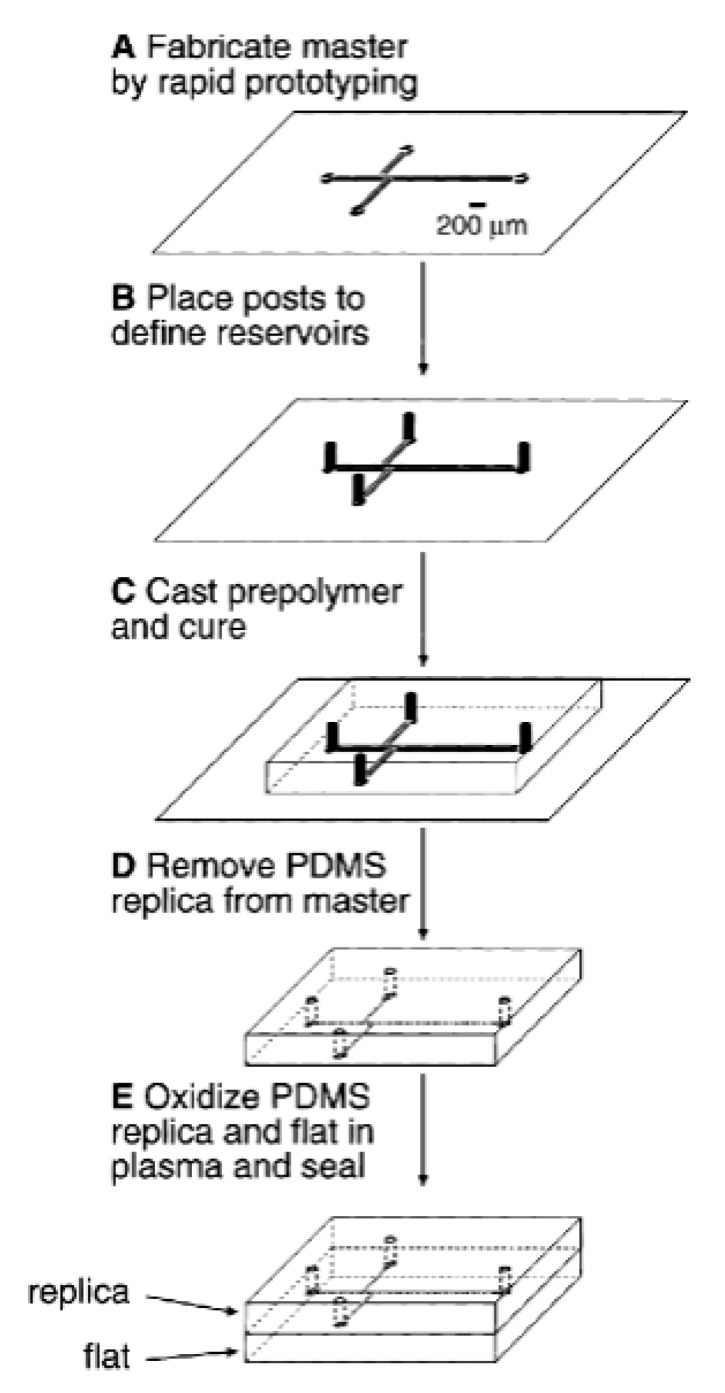
Schematic representation of micro-channel fabrication using soft lithography. (**A**) A high-resolution transparency containing the design of the channels, created in a CAD program, was used as the mask in photolithography to produce a positive relief of photoresist on a silicon wafer. The scale bar gives an indication of the thickness and width of photoresist. (**B**) Glass posts were placed on the wafer to define reservoirs for analytes and buffers. (**C**) A prepolymer of PDMS was then cast onto the silicon wafer and cured at 65 °C for 1 h. (**D**) The polymer replica of the master containing a negative relief of channels was peeled away from the silicon wafer, and the glass posts were removed. (**E**) The PDMS replica and a flat slab of PDMS were oxidized in a plasma discharge for 1 min. Plasma oxidation had two effects. First, when two oxidized PDMS surfaces were brought into conformal contact, an irreversible seal formed between them. This seal defined the channels as four walls of oxidized PDMS. Second, silanol (SiOH) groups introduced onto the surface of the polymer ionize in neutral or basic aqueous solutions and support EOF in the channels. Taken with permission from [131].

Soft lithography is a conventional method for fabricating microfluidic devices from soft polymers such as PDMS. Master prototyping followed by replica moulding is often an effective and cost-efficient technique to fabricate elastomeric devices with high fidelity [129,130]. A master template of silicon or glass is created by different techniques, although the standard method uses photolithography [103]. To fabricate a device a liquid pre-polymer is cast over a master template. It is typical to silanise the master template to prevent the polymer adhering to the master post curing. Upon curing the liquid prepolymer becomes elastomeric in nature and can be peeled from the master mould to leave a replica of the master features in the cured elastomer [102,131]. Figure 15 describes the steps involved in fabricating a microfluidic device using master prototyping and replica moulding. After casting the PDMS device, sealing is carried out by treating the PDMS device using oxygen plasma which will form an irreversible bond with the sealing material. PDMS is a flexible polymeric material that can seal to different substrates, either reversibly or irreversibly, depending on the application [131].

In one application, a brass mould was used to cure PDMS to fabricate a 2.5mm thick device with a central hole. This device was further treated with oxygen plasma to achieve a permanent bonding with a glass coverslip to enhance the acoustic contact when placed over the surface acoustic wave (SAW) transducer. The novel device uses the principle of SAW-induced mixing of sample blood with assay reagents including fluorescent microspheres. The clotting time was derived from the time taken by the rapid tumbling of the fluorescent microspheres to cease as the clot formation progresses [132].

A microfluidic haemostasis model consisting of eight inlets and a single outlet channel was cast in PDMS using soft lithography. The PDMS device was reversibly sealed to the collagen patterned glass slide using vacuum bonding. The device was then exposed to corn trypsin inhibitor-treated whole blood under venous blood flow conditions. This device demonstrated thrombus formation in the presence of collagen and no additional TF, emphasizing the study of contact pathway-dependent blood coagulation and fibrin deposition in haemophilic diseases [70].

### 3.3. Additional Consideration – Biomolecule Patterning

Biomolecule patterning is a technique for immobilizing biomolecules to a substrate. One of the common methods for patterning proteins within a specific location on microfluidic devices is deep UV photolithography. The technique was adopted from the field of microelectronics where it was initially used to develop bio-electronic circuits. Multiple biomolecules can be either physically adsorbed or chemically bonded on the solid support to form a biomimetic model [133]. Deep-UV irradiation, along with silanisation, modifies the selected layers of the substrate on which a protein binds without altering its function. There are several direct printing methods where the printing pin comes in contact with the substrate to deposit the biomolecules. Barbulovic-Nad et al. gave an extensive description of each of the methods available for biomolecule patterning [134].

**Figure 16:**
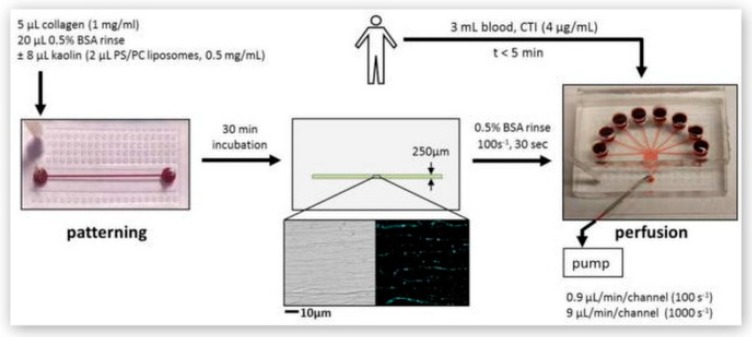
Experimental set-up: Collagen/kaolin patterned to a glass slide with a thickness of 250 µm. The microfluidic device with 8 inlets was then bonded to the glass slide. Taken with permission from [135].

Shen et al. formed silica micro-capillaries using deep-UV photolithography to study coagulation at different blood flow rates. The deep-UV light was used to remove selected layers in a silanised capillary and fill it with a 200 µm thick layer of tissue factor (TF). This capillary-based microfluidic device demonstrated the importance of TF in the initial phase of coagulation and the effect of shear stress on TF activity [72]. Figure 16 illustrates another microfluidic device patterned with two blood clot initiating proteins (collagen and kaolin) which was developed for studying contact activation of blood coagulation. The protein patterning device was fabricated using PDMS and consisted of a single channel. This device was vacuum bonded onto a glass slide and 5 µL of the collagen sample was injected into the microchannel. Thus, collagen formed a thin layer over the glass slide, over which the fluorescently labelled kaolin was coated. The concentration of collagen/kaolin covering the surface of the device was evaluated using the Image J software based on the fluorescence intensity [135] and the contact activation of clotting in a controlled microfluidic device analysed.

## 4. Commercially Available Devices

POC devices have significantly increased the quality of clinical care in terms of rapid diagnostic test results, increased flexibility, and reduced laboratory processing time. The global market share of POC devices is expected to reach USD 38.1 billion by 2022, with a compound annual growth rate of 10%, of which majority of the devices are based on lateral flow assays [136]. With advancements in microfluidic technology, LOC-based research is progressing to develop next-generation POC devices. Chin et al. have descriptively summarised the market analysis of microfluidic POC devices and listed the companies that have successfully developed microfluidic devices for blood biochemistry analysis, immunoassays, cardiac biomarkers, infectious disease tests, pregnancy-related analysis, and others [137]. Table 1 outlines the features and detection methods for the commercially available devices reviewed in this section.

Coumatrak by Biotrack (Fremont, CA, USA) and Ciba Corning Biotrack 512 (Medfield, MA, USA) were among the first-generation coagulation (PT/INR) monitoring devices used in near-patient settings. The devices were comprised of a monitor with an optical detection system and a test cartridge with a channel through which the whole blood sample is drawn to the underlying dried reagent via capillary action. The values of PT and INR were displayed on the monitor based on the clotting time, and the devices were widely used in patient self-management of warfarin [138,139]. The second-generation POC devices for monitoring PT/INR were designed by Roche Diagnostics (Basel, Switzerland) and included the CoaguChek series, of which CoaguChek and CoaguChek S were replaced with the advanced version CoaguChek XS, a small-sized handheld device. This device works on the principle of electrochemical detection in which the clotting time is derived from the electrical signal generated by an electrochemical agent released in the presence of thrombin at the time of clotting. The CoaguChek XS system also incorporates an integrated quality check feature to ensure the integrity of each test strip in the event of exposure to high temperature, light or humidity [140]. The iSTAT^®^PT/INR (Abbott Laboratories, Chicago, III, USA) system works on the same electrochemical detection principle and consists of a microfluidic test cartridge integrated with sensors. In one study conducted at an anticoagulation clinic, PT/INR values of 52 patients on warfarin when measured using iSTAT PT/INR and CoaguChek XS Plus were compared with the STAGO system, a laboratory-based coagulation analyser. The results obtained from this small population study demonstrated that CoaguChek XS Plus is more accurate, but both the devices displayed certain deviation from standard laboratory values [141]. The electrical impedance-based detection method was employed in the INRatio/INRatio2^®^ (Alere Inc., Waltham, MA, USA) system for PT/INR monitoring but the device was recalled by the manufacturer in 2016 as it gave a significantly lower INR value than the laboratory measurements [142].

The ITC ProTime^®^ Microcoagulation (International Technidyne Corp (ITC), Edison, NJ, USA) system is a handheld battery-operated PT/INR monitoring system comprised of cuvettes with micro-channels, an optical detection system with an array of LEDs and an integrated quality control system. The INR value is obtained from the clotting time derived by detecting the cessation of the blood flow with the progression in clot formation in the micro-channel. A multicentre study demonstrated that the accuracy of ProTime^®^ Microcoagulation system is equivalent to the standard laboratory plasma test [143]. The Coag-Sense™ PT/INR Monitoring System (CoaguSense, Inc., Fremont, CA, USA) employs direct clot technology and has a unique combination of test strips with wheel-like appearance and optical-mechanical mode of detection that uses a laser beam on the periphery of a rotating wheel. In one single-centre study, two novel POC devices, MicroINR^®^ (iLine Microsystems S.L, Gipuzkoa, Spain) and ProTime InRhythm™ (ITC, Edison, NJ, USA), were used to demonstrate the INR accuracy along with existing POC devices (CoaguChek XS and INRatio2 system). MicroINR^®^ (iLine Microsystems S.L, Gipuzkoa, Spain) is a hand-held PT/INR monitoring device designed for professional use, self-testing, and self-management. The device consists of a portable analyser, disposable test chip comprising of two micro-channels one a reaction chamber, and the other an internal control system. A POC device specifically designed for PT/INR and based on pressure-dependent detection is the ProTime InRhythm™ (ITC, Edison, NJ, USA). The test cuvettes include micro-channels where clot detection is performed in duplicate. The blood from the sample chamber is drawn into the micro-channels, where it is mixed with the reagents through continuous pumping and increasing the pressure on the channel with the progression of clot formation [144]. A few other POC devices for PT/INR monitoring are CoagLite^®^ and CoagMax^®^ (Microvisk Technologies, Saint, Asaph, UK) and Bio-AMD’s COAG (Bio- Alternative Medical Devices Ltd., Warrington, UK) [145]. In 2016, Xprecia Stride (Siemens Healthineers, Erlangen, Germany), a new handheld PT/INR device was given clearance by FDA. In a recent study, the Xprecia Stride was compared to the standard laboratory method and the CoaguChek POC device (ITC, Edison, NJ, USA). The PT/INR resulted in a correlation of 87% with the laboratory method and 93% with CoaguChek devices within 0.5 INR units of each other [146].

The Hemochron series (ITC, Edison, NJ, USA) of automated coagulation monitoring systems have long been available for regulating high doses of heparin during cardiac surgeries. The first generation of Hemochron devices used for measuring ACT was based on the mechanical model of detection in which the clotting time was derived from the time of the coagulation-induced displacement of a magnet in the test tube. The newer generation devices (Hemochron Jr^®^ series) are based on optical detection, employing a series of LEDs and long-range cuvettes such as ACT+ and ACT-LR [21]. Medtronic (Minneapolis, MN, USA) offers two coagulation systems: the haemostasis management system (HMS plus) and the automated coagulation timer system (ACT plus). HMS plus/kaolin-ACT system is designed to measure the heparin concentration by means of a heparin dosage response (HDR) assay and an optical detection technique. In a recent study, the concentration of heparin in post-reperfusion circulating blood after the liver transplantation obtained using HMS plus was compared against the laboratory standard anti-Xa assay. The results were significantly positive, inferring that the laboratory anti-Xa assay-dependent heparin measurement could be replaced with the HMS plus system [147]. iSTAT (Abbott Laboratories, Chicago, III, USA) is one of the most successful multi-test analysers, offering a range of blood chemistry tests, cardiac markers and coagulation tests, including PT/INR and ACT [148].

Helena Laboratories POC (Beaumont, Tex, USA) offers several POC devices, including the Cascade POC analyser, Plateletworks^®^, and the Actalyke^®^ family for monitoring antithrombotic drugs, performing coagulation tests as well as platelet aggregation studies. The cascade POC analyser can perform major coagulation tests such as ACT, PT, and aPTT. This system is comprised of test cards with paramagnetic iron oxide particles and a unique combination of a photodetector and an electromagnet to analyse the clotting time. Upon application of the blood sample to the reaction chamber in the test card, it reacts with the reagent and paramagnetic particles. The electromagnet-induced movement of paramagnetic particles will gradually decrease as the clot formation progresses, which is detected optically. A photodetector collects the data of the light reflected based on the displacement of paramagnetic particles, which are subsequently converted to the clotting time [149]. Actalyke XL, the next generation ACT system, has two points clot detection technique, long measurement range, and battery back-up. Alongside this, Helena Laboratories also provide a range of ACT assay test tubes that are designed to be compatible with other devices such as the Hemochron series [21]. Instrumentation Laboratory’s (Bedford, MA, USA) multi-test device (GEM^®^PCL Plus) is a portable whole blood coagulation analyser that provides rapid results for PT/INR, aPTT, and ACT.

Platelet function testing (PFT) can be utilized to study platelets by monitoring platelet dysfunction, bleeding conditions, haemorrhagic disorders, perioperative haemostasis, and antiplatelet treatment. Platelet aggregometry based on light transmission (LTA) is a gold standard method for PFT, but it is time consuming and requires platelet-rich plasma samples. The POC devices such as Multiplate^®^ analyser (Multiple function platelet analyser, Roche Diagnostics, Basel, Switzerland) and PlateletWorks^®^ (Helena Laboratories, Beaumont, Tex, USA) use whole blood assays based on impedance aggregometry and platelet reactivity [150]. The Multiplate^®^ analyser consists of electrodes integrated with five test channels that monitor the change (increase) in impedance observed as a consequence of platelet aggregation. This multiple electrode aggregometry analyser proved to be a sensitive test for verifying platelet dysfunction induced during extracorporeal circulation [151]. PlateletWorks^®^ monitors platelet activity by implementing Coulter counting to measure the platelet count pre and post aggregation. The ratio of the platelet count in the control tube with ethylenediaminetetraacetic acid (EDTA) to the aggregation activating citrated tube demonstrates the platelet adhesion and aggregation in a rapid manner [149]. The PFA-100/200 (Siemens Corporation, Malvern, PA, USA) series platelet function analyser has been on the market for more than 20 years and is used for the diagnosis of Von Willebrand Disease (VWD) and other platelet disorders [152]. Shear stress-dependent platelet adhesion study can be carried out by a cone and plate analyser where a rotating cone will generate uniform shear stress on the sample over the plate. The IMPACT-R (Cone and Plate(Let) Analyzer (CPA), DiaMed, Cressier, Switzerland) technology is beneficial in screening bleeding disorders and monitoring antiplatelet therapy while utilizing only 130 µL of citrated whole blood [153]. A unique combination of optical detection- and electromagnetic-induced mixing of samples and reagents is combined in the VerifyNow system (Accumetrics, San Diego, CA, USA). This device consists of an assay cartridge containing a platelet agonist and fibrinogen beads (a platform for platelet adhesion) for monitoring antiplatelet therapy. POC devices designed for examining the viscoelastic properties of whole blood include TEG^®^ (Haemonetics Corp., Braintree, MA, USA), ROTEM^®^ (Pentapharm GmbH, Munich, Germany) and Sonoclot Analyser (Sienco Inc., Boulder, CO, USA), which are widely used to obtain a detailed analysis of clot formation and lysis in clinical setting [154,155,156].

The rise in oral antithrombotic therapy has intensified the development of innovative approaches to monitoring the therapy. The ability to perform global haemostasis tests such as PT, aPTT, and ACT in near-patient settings has improved clinical management. In the case of trauma, PFT and viscoelastic POC devices have proved be significant aids in rapidly assessing the coagulation state [156,157]. With the introduction of next-generation technology, the accuracy and precision of POC devices is continuously being improved and enhanced.

## 5. Conclusion and Future Perspective

Microfluidics is now established as a distinct technological field, although the development of microfluidic devices only began circa 40 years ago. LOC technology has made it possible to carry out a total analysis which includes sample preparation, chemical interactions, fluidic transport and detection in one device within micrometer dimensions. These advantages have increased the application of microfluidics in the biological diagnostics world. Along with this, the controlled environment of micro-devices offers the ability to change the fluidic parameters, making it possible to study the different stages of the thrombotic condition. For example, the changes in flow rate and pressure at varied geometric dimensions mimicking the vascular haemodynamic circumstances have led to the understanding of the role of various factors responsible for platelet aggregation during thrombosis.

The flexibility in developing the micro-channels of capillary sizes is also the result of advancements in new materials and fabrication technologies. The possibility of introducing multi-channel reaction chambers onto a single microchip is imperative in developing high throughput devices. This has also contributed to fabricating devices that can conduct multiple parallel tests simultaneously, using the sample in microlitre volumes. In addition to this, the method of patterning the desired protein onto a material substrate has widened the research of haemostatically active proteins and their role in the coagulation pathways. The adaptation of microelectronics fabrication methods in microfluidics has made it possible to incorporate electrodes and sensors into the same device, further miniaturising the device. Small diameters, accommodating low volumes of blood samples and reagents, have decreased both the cost and the timing of experiments compared to conventional laboratory methods.

As the microfluidic field is multidisciplinary and still in its growing phase, there are certain challenges faced by researchers. One of the limitations in developing biomimetic vascular models is maintaining the constant shear rate throughout the micro-channel due to its rectangular geometric design and fabrication [158]. This could be addressed by changes in design, such as creating circular micro-channels instead of rectangular [159], which is expected to develop with the future advancements in fabrication technologies. A multitude of assays are available for monitoring antithrombotic therapies but incorporating the assay into a microfluidic device is a significant challenge. The major concerns lie in the miniaturisation and transport of fluid within the micro-channels while maintaining the sensitivity and specificity of the coagulation test.

The future expectation from microfluidic technology is the commercialisation of next generation devices. Commercialisation in this space will depend upon factors such as ease of use, low cost and reliability in terms of sensitivity and specificity. If these goals are achieved, there is a greater likelihood of the growth of low-cost high-throughput devices which will provide rapid results in near-patient settings in the field of haemostasis.

## Figures and Tables

**Table 1:** Features detection methods and for a selection of commercially available coagulation monitoring devices.

PT/INR Monitoring Devices		
Device	Features	Detection Method
Coumatrak (Biotrack, USA)	First Generation Device	Optical
Ciba Corning Biotrack 512 (Ciba Corning Diagnostics, USA)	First Generation Device	Optical
CoaguChek Series (Roche Diagnostics, Switzerland)	Second Generation small-sized handheld Devices Integrated with test strip quality check feature	Electro-chemical
iSTAT^®^ PT/INR (Abbott Laboratories, USA)	Test cartridge integrated with sensors	Electro-chemical
INRatio/INRatio^®^2 (Alere Inc., USA)	Recalled in 2016 for erroneous results	Electro-mechanical (Electrical Impedance)
ProTime^®^ Microcoagulation (International Technidyne Corp, USA)	Test cuvettes with micro-channels Integrated quality control system	Optical
Coag-Sense™ PT/INR Monitoring System (CoaguSense, Inc., USA)	Direct clot detection technology	Optical-mechanical
MicroINR^®^ (iLine Microsystems S.L, Spain)	Test chip with one inlet and two micro-channels (Control and PT time test channel)	Optical
ProTime InRhythm™ (International Technidyne Corp, USA)	Pressure-driven clot formation and detection microchannel cuvette	Mechanical
CoagLite^®^ and CoagMax^®^ (Microvisk Technologies, UK)	MEMS-base	Electro-mechanical
Bio-AMD’s COAG (Bio-Alternative Medical Devices Ltd., UK)	Optical-magnetic mode of detection	Optical
Xprecia Stride™ Coagulation System (Siemens Healthineers, Germany)	Test strip consists of an electrochemical cell	Electro-chemical
**ACT/aPTT Monitoring Devices**		
**Device**	**Features**	**Detection Method**
Hemochron Series (International Technidyne Corp, USA)	Test method – Test tube containing magnet and Test cuvettes preloaded with celite/kaolin/silica	Mechanical (First generation devices) Optical (Second generation devices)
HMS plus and ACT plus (Medtronic, USA)	Heparin Dosage Response assay	Optical
iSTAT (Abbott Laboratories, USA)	Multi-test analyser including PT/INR test with ACT and aPTT	Electro-chemical
Cascade POC and Actalyke^®^ series (Helena Laboratories POC, USA)	Cascade POC - Paramagnetic particles-based assay Actalyke XL – Two-point clot detection technique	Optical-Mechanical
GEM^®^ PCL Plus (Instrumentation Laboratory, USA)	Multi-test device for PT/INR, ACT and aPTT monitoring	Optical
**Platelet Function Analysers**		
**Device**	**Features**	**Detection Method**
Multiplate^®^ (Roche Diagnostics, Switzerland)	Impedance aggregometry and platelet reactivity assays	Electromechanical (Electrical Impedance)
PlateletWorks^®^ (Helena Laboratories POC, USA)	Platelet aggregation and platelet reactivity monitoring based on platelet counts	Electromechanical (Electrical Impedance)
PFA-100/200 (Siemens Corporation, USA)	Flow based assay system	Mechanical
IMPACT-R Cone and Plate (Let) Analyser (DiaMed, Switzerland)	Shear stress dependent platelet adhesion monitoring	Optical
VerifyNow (Accumetrics, USA)	Electromagnetic induced mixing of sample and reagents	Optical
TEG^®^ (Haemonetics Corp., USA) ROTEM^®^ (Pentapharm GmbH, Germany) Sonoclot Analyser (Sienco Inc., USA)	Detailed Analysis of blood clot formation and lysis based on viscoelastic properties of blood	Mechanical
